# Staphylococcal Enterotoxins Modulate Platelet Response During Storage of Platelet Concentrates and Impair Silkworm Survival

**DOI:** 10.3390/toxins17120593

**Published:** 2025-12-11

**Authors:** Sylvia Ighem Chi, Chelsea McGregor, Nicolas Pineault, Sandra Ramirez-Arcos

**Affiliations:** 1 Medical Microbiology, Canadian Blood Services, Ottawa, ON K1G 4J5, Canada; 2 Department of Biochemistry, Microbiology and Immunology, University of Ottawa, Ottawa, ON K1G 4J5, Canada; 3 Research, Canadian Blood Services, Ottawa, ON K1G 4J5, Canada

**Keywords:** cytokine release, host–pathogen interaction, miRNA profiling, platelet activation, platelet concentrates, silkworm infection model, *Staphylococcus aureus*, staphylococcal enterotoxin

## Abstract

Platelet concentrates (PCs) are used to treat patients with platelet deficiencies. PCs are stored at 20–24 °C under agitation for up to 7 days to maintain platelet functionality, but these conditions are amenable for proliferation of contaminants such as *Staphylococcus aureus*, posing a risk for transfusion-transmitted infections. We investigated the contribution of staphylococcal enterotoxins (SEs) type G (SEG) and type H (SEH) to platelet activation, cytokine release, microRNA (miRNA) modulation, and in vivo virulence. PCs were inoculated with wildtype *S. aureus* CBS2016-05 or SE-deficient mutants (Δ*seg*, Δ*seh*, ΔΔ*segh*) and monitored during storage. Flow cytometry revealed progressive elevation of platelet activation markers CD62P and Annexin V in contaminated PCs, with significantly higher expression in wildtype compared to SE-mutant strains. Cytokine profiling demonstrated that SEs modulate pro- and anti-inflammatory mediators, notably CCL2, TGF-β1, IFN-γ, and TNF-α, implicating SEG in their regulation. Next-generation sequencing and RT-qPCR validation identified transient induction of immune-related microRNAs miR-98-5p, miR-146a-5p, miR-221-3p, miR-320a-3p, with SE-dependent expression patterns. In a silkworm infection model, wildtype *S. aureus*-contaminated PCs exhibited significantly higher lethality than SE-deficient strains, confirming toxin-mediated virulence. Collectively, these findings reveal that SEs exacerbate platelet activation and immune dysregulation during storage, enhancing bacterial pathogenicity. This study identifies platelet-derived cytokine and miRNA signatures as potential biomarkers of bacterial contamination and underscores the need to mitigate SE-driven platelet dysfunction to improve transfusion safety.

## 1. Introduction

Platelet concentrates (PCs) are critical transfusion products in hemostatic support, but they remain vulnerable to bacterial contamination, which can lead to transfusion-transmitted infections and compromise PC safety [1]. Among PC contaminants, *Staphylococcus aureus* is frequently isolated during routine screening and can cause fatalities [2,3,4,5,6,7]. *S. aureus* is known to interact with platelets, triggering activation and aggregation via both direct binding and indirect coagulation pathways (through fibrinogen bridges) [8,9,10]. The activation of platelets by *S. aureus* in contaminated PCs during storage has been reported [11]. However, the specific virulent modulators are yet to be uncovered.

Superantigenic toxins, such as those produced by *S. aureus*, represent virulence factors that may further modulate host immune responses and cellular physiology, but their role in platelet–bacterium interactions in the unique milieu of stored PCs is poorly understood. The *S. aureus* superantigen, toxic shock syndrome toxin 1 (TSST-1) has been reported to influence platelet function [12]. However, the specific effects of staphylococcal enterotoxins (SEs) remain understudied, particularly in the context of PC contamination. We have previously demonstrated that *S. aureus* can secrete exotoxins including SEs during storage of PCs [13,14]. SEs are superantigens capable of inducing massive cytokine release through non-specific activation of T lymphocytes [15]. Although classical SEs (e.g., SEA, SEB) are widely recognized for their roles in *S. aureus* virulence, several non-classical enterotoxins, including SEG and SEH, are increasingly detected among clinical isolates and have been implicated in immune activation and disease severity [16,17]. Previously, our work has focused on the mechanisms of adaptation employed by *S. aureus* in the immune challenging PC milieu, and this present study aimed to investigate how platelets respond to *S. aureus* virulence, especially the exposure to SEs in the PC bag. Whole-genome sequencing of *S. aureus* CBS2016-05, a PC–associated clinical isolate, has confirmed that it encoded enterotoxins, *seg* and *seh*, and lacks the classical SE genes [18]. Furthermore, our previous study demonstrated that SEG and SEH are secreted and detectable in PCs during storage [19]. The rationale for choosing *S. aureus* CBS2016-05 as well as SEG and SEH for the present study is based on the above findings from previous work, which provide direct evidence that these toxins are secreted and stable in the PC storage milieu. This observation allowed us to interrogate platelet responses to SEs that are not only clinically relevant but also experimentally validated under PC storage conditions. Moreover, because SEG and SEH remain comparatively understudied relative to the classical SEs, examining their specific contributions offers an opportunity to expand our understanding of how diverse SE families modulate platelet activation and inflammatory pathways. While SEs have been extensively studied in the context of immune activation, their effects on platelet function remain underexplored. Platelets are increasingly recognized as immune cells, participating not only in hemostasis but also in innate and adaptive immunity through secretion of cytokines, expression of adhesion molecules (such as P-selectin), and release of extracellular vesicles [20]. There is evidence showing that bacterial toxins modulate platelet responses, influencing thrombo-inflammation, and sepsis outcomes [21].

MicroRNAs (miRNAs) are emerging as sensitive regulators of platelet biology and mediators of immune signaling responses in sepsis and infectious inflammation [22,23]. Platelet-derived miRNAs regulate post-transcriptional gene expression in vascular and immune contexts, with miR-221 and miR-146a being particularly linked to inflammatory signaling [24,25]. Annexin-V binding is widely used as a marker of platelet activation and apoptosis-like events [26]. However, the contribution of SEs to platelet miRNA and platelet activation marker expression is not well characterized.

Silkworm (*Bombyx mori*) larvae provide a practical in vivo model for studying *S. aureus* virulence because its conserved innate immune pathways, including Toll signaling, hemocyte-mediated responses, and antimicrobial peptide production, parallel mammalian innate immunity [27,28]. Although it lacks adaptive immunity, the model enables controlled, high-throughput assessment of bacterial virulence and toxin effects before mammalian studies. Silkworms have been successfully used to assess the pathogenicity of *S. aureus* toxins [28], yet the specific role of SEs in host lethality remains insufficiently investigated. This study aimed to examine the effects of SEG and SEH on (1) surface expression of P-selectin, (2) cytokine secretion from platelets, (3) platelet miRNA profiles, and (4) *S. aureus* virulence in silkworms. We hypothesized that SE exposure would induce a pro-inflammatory phenotype in platelets and impair silkworm survival in a *S. aureus* strain-dependent manner.

## 2. Results

### 2.1. Staphylococcus aureus Heightens Platelet Activation During Storage of S. aureus-Contaminated PCs

**Figure 1:**
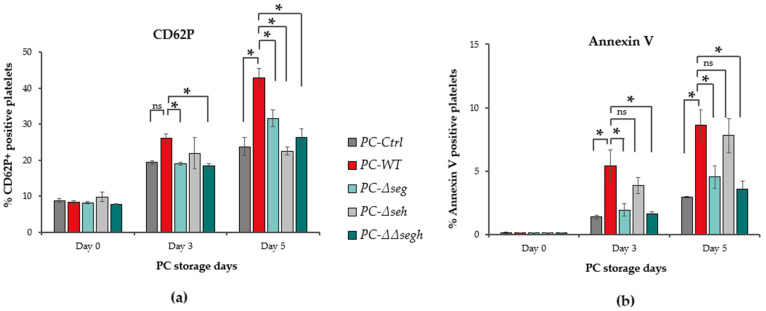
Expression of platelet activation markers increases over time during PC storage. (**a**) CD62P (**b**) Annexin V are heightened in *S. aureus* inoculated PCs. However, a decrease is observed in SE mutants compared to WT spiked PCs. Error bars; SEM. Significant if * *p* ≤ 0.05, ns; non-significant, *n* = 3.

The expression of platelet activation markers CD62P (P-selectin) and Annexin V were assessed in PCs contaminated with wildtype *S. aureus* CBS2016-05 (PC-WT) and corresponding toxin deficient strains (PC-Δ*seg*, PC-Δ*seh* and PC-ΔΔ*segh*) in comparison with non-spiked PC (PC-Ctrl), tested at days 0, 3 and 5 of PC storage. Flow cytometric data revealed the following: (1) progressive increased expression of CD62P and Annexin V markers from days 0 to 5 in all test samples (Figure 1). (2) There were no statistical differences at day 0 for both markers, as well as day 3 for CD62P (~0.5 fold, *p* = 0.06) (Figure 1a); however, Annexin V had increased expression in PC-WT compared to PC-Ctrl at day 3 (~1.8 fold, *p* = 0.022) and day 5 (*p* = 0.03 and 2.2 fold, *p* = 0.04) (Figure 1b). (3) When comparing PCs inoculated with PC-WT and its SE-mutant counterparts (PC-Δ*seg*, PC-Δ*seh* and PC-ΔΔ*segh*), a significant decrease was observed for CD62P (Figure 1a) and Annexin V (Figure 1b) in the SE-mutant spiked PCs compared to PC-WT for all (*p* < 0.05) except PC-Δ*seh* at day 3 (*p* = 0.07). These results are indicative of increased platelet activation and procoagulant activity over time during PCs storage. Importantly, the presence of *S. aureus* in PCs can exacerbate the activation of platelets during storage. Furthermore, the observed decline of activation markers in PCs contaminated with enterotoxin deficient strains show that these toxins play a key role in platelet activation.

### 2.2. Staphylococcal Enterotoxins Exacerbate a Pro-Inflammatory Profile in Platelets During PC Storage

**Figure 2:**
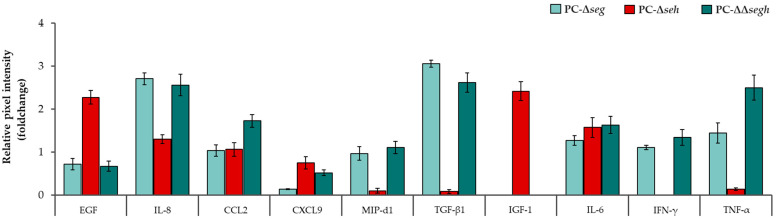
Release of inflammatory modulators in SEs mutants compared to WT-spiked PCs. SEH potentially influence expression of EGF and IGF-1, while SEG plays a key role in the release of MIP-d1, TGF-β1, IFN-g, TNF-a. IL-6, IL-8, (Interleukins 6, 8), IFN-g (interferon-gamma), TNF-α (tumor necrosis factor α), CCL2 (C-C class chemokine 2), CXCL9: (C-X-C motif ligand 9), MIP-d1 (Macrophage Inflammatory Protein-1 delta), EGF (Epidermal growth factor), TGF-β1 (Transforming growth factor β). Error bars; SEM. Significant *p* ≤ 0.05, *n* = 3.

Platelet concentrate samples inoculated with PC-WT and derivative toxin mutant strains (PC-Δ*seg*, PC-Δ*seh* and PC-ΔΔ*segh*) were evaluated in comparison with PC-Ctrl at days 0, 2 and 3 for cytokine production using an array dot membrane cytokine kit. Densitometric analyses revealed an overall storage time-dependent increase in cytokine production in PCs as most expressed cytokines were abundant at day 3 compared to day 0. Interestingly, the exposure of platelets to PC-WT increased secretion of the anti-inflammatory cytokines CCL2 (C-C class chemokine 2), EGF (Epidermal growth factor), OPN (Osteopontin) and TGF-β1 (Transforming growth factor β1), and pro-inflammatory mediators IL-8 (Interleukin 8), MIP-3α (Macrophage inflammatory protein-3α), IFN-γ (Interferon γ) and TNF-α (Tumor necrosis factor α) as opposed to the background PC-Ctrl, at days 2 and 3 of storage (Table 1). The following observations were noted when investigating cytokine production in PC-Δ*seg*, PC-Δ*seh* and PC-ΔΔ*segh* with PC-WT as baseline at day 2 of PCs storage: (1) Both anti-and pro-inflammatory cytokines were elevated in all PCs spiked with SE-deficient strains compared to the wildtype (Figure 2). (2) The single (Δ*seg*) and double mutant (ΔΔ*segh*) inoculated PCs show similar expression profiles for most of the differentially expressed cytokines. (3) Figure 2 shows some discrepancies between the single mutants Δ*seg* and Δ*seh* contaminated PCs: while EGF and IGF-1 were more than two-fold increased in PC-Δ*seh* relative to PC-WT, a similar trend was observed for IL-8 and TGF-β1 in PC-Δ*seg*. (4) Importantly, four pro-inflammatory mediators (MIP-d1, TGF-β1, IFN-γ, TNF-α) were significantly reduced in PC-Δ*seh* compared to PC-Δ*seg*. (5) Additionally, IGF-1, which had elevated levels in the PC-Δ*seh*, is not impacted in both PC-Δ*seg* and PC-ΔΔ*segh* samples. These results indicate that the secretion of different SEs in PCs during storage, individually or collectively, lead to the release of numerous paracrine factors including cytokines and chemokines from platelets. SEG likely plays a key role in the secretion of MIP-d1, TGF-β1, IFN-γ and TNF-α, while SEH influence expression of IGF-1 and EGF.

### 2.3. Platelet microRNA Profile Is Altered by Staphylococcal Enterotoxins During PC Storage

**Figure 3:**
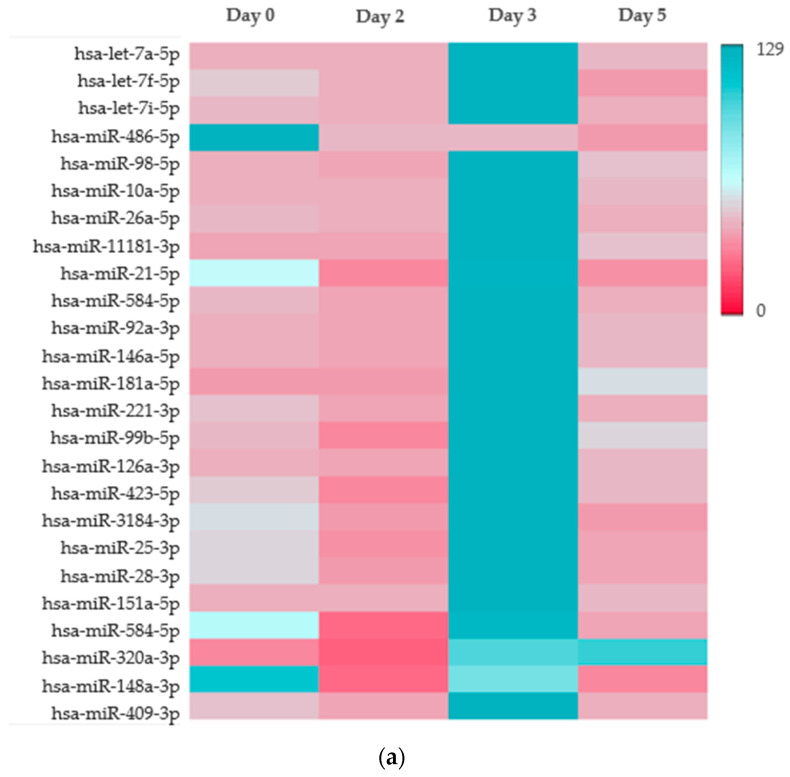

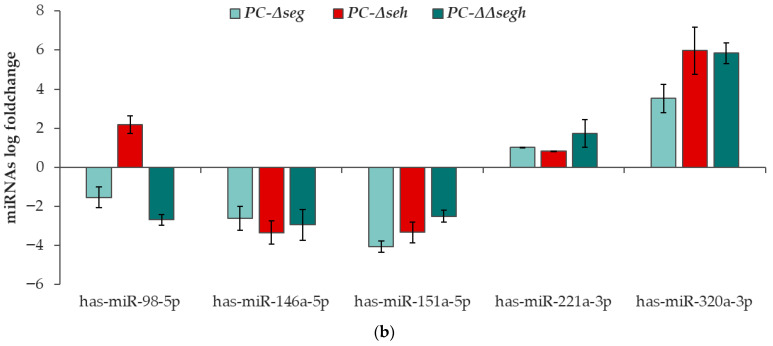
(**a**): Heatmap showing 25 differentially expressed miRNA in *S. aureus* spiked platelet concentrates (PC-WT) in comparison to non-spiked control (PC-Ctrl). (**b**): RT-qPCR evaluation of differentially expressed miRNAs (hsa-miR-98-5p, hsa-miR-146a-5p, hsa-miR-151a-5p, hsa-miR-221a-3p and hsa-miR-320a-3p) in PCs contaminated with SEs deficient mutant strains (PC-Δseg, PC-Δseh and PC-ΔΔsegh) versus PCs spiked with the wildtype strain (PC-WT) in comparison to non-spiked control (PC-Ctrl). Error bars; SEM. Significant *p* ≤ 0.05, *n* = 3.

Platelet miRNAs extracted from days 0, 2, 3 and 5 PCs that were inoculated with PC-WT were sequenced together with the corresponding non-contaminated controls (PC-Ctrl). The miRNAseq data revealed the following: Firstly, the miRNAseq data show overall stable expression of the let-7 family members (let-7a and let-7f) and hsa-miR-486-5p, which were consistently among the topmost 15 expressed miRNAs in PC-Ctrl as well as PC-WT throughout storage, indicating baseline or constitutive expression in PCs (Figures S1 and S2). Secondly, specific miRNAs including miR-221, miR-26a, miR-98, and miR-21 showed marked increases in expression in PC-WT, particularly on day 3 (Figure S2 Left), suggesting activation in response to bacterial contamination or storage-associated stress. While the line plots (Figure S1 Right) indicate a sharp increase in several miRNAs during storage of PC-Ctrl (notably let-7 family members and miR-486-5p), graph plots of spiked PCs (Figure S2 Right) revealed remarkable upregulation of the same miRNAs peaking on day 3, followed by a decline by day 5, implying a transient response phase. Thirdly, differential expression analysis of various miRNAs in *S. aureus* spiked versus non-spiked PCs reveal a distinct upregulation of multiple miRNAs including miR-98, miR-151a, miR-146a and miR-320a on Day 3 (Figure 3a, Table S1). It is worth noting that the sharp increase is transient as expression levels return close to baseline level by Day 5, suggesting a time-specific miRNA response. Some miRNAs such as miR-486, miR-409, and miR-148a also showed higher expression at Day 0, indicating elevated baseline levels or early response.

Five differentially expressed miRNAs (miR-98, miR-146a, miR-151a, miR-221, and miR-320a) were selected based on their known immune regulatory roles in infections for RT-qPCR validation. Levels were compared between PCs contaminated with wildtype and SE-deficient strains (Figure 3b). RT-qPCR data revealed significant upregulation of hsa-miR-320a-3p (~3 to 6 folds) in all SE-mutant spiked PCs and miR-221a (~1.8 fold in PC-ΔΔ*segh*) compared to PC-WT, while miR-98 was only elevated in PC-Δ*seh*, indicating that SEH plays a role in modulating these miRNAs and that both SEG and SEH may play keys in the regulation of miR-320a during platelet contamination. On the contrary, miR-146a and miR-151a were significantly downregulated in all mutant-contaminated PCs (~−4.0 to −2.1 folds) and miR-98 was decreased in both PC-Δ*seg* and PC-ΔΔ*segh* compared to PC-WT. These results confirm the impact of SEs on expression levels of miRNA in PC.

### 2.4. Staphylococcal Enterotoxins Increase Silkworm Mortality

Fifth-instar silkworm larvae injected with PCs inoculated with wildtype or SE-mutant *S. aureus* strains showed strain-dependent mortality. The LD_50_ value for the wildtype strain was approximately 3.31 × 10^6^ CFU/larvae, 1-log lower than the LD_50_ values obtained for the SE-deletion mutants PC-Δ*seg*, PC-Δ*seh* and PC-ΔΔ*segh*, (approximately 2.30 × 10^7^, 3.50 × 10^7^ and 8.90 × 10^7^ CFU/larvae, respectively) (Table 2), which indicates that these SEs play an essential role the virulence of *S. aureus* in a silkworm model. Furthermore, the virulence of these bacterial strains in contaminated PCs resulted in high mortality rate in injected larvae with only 27% survival for PC-WT compared to 37% for PC-Δ*seg*, 30% for PC-Δ*seh* and 40% for PC-ΔΔ*segh,* respectively (Table 2). Significantly higher silkworm survival rates were observed in PC-ΔΔ*segh* in comparison to PC-WT (*p* = 0.026, Table 2).

## 3. Discussion

In this study, we investigated the contribution of SEG and SEH to platelet responses within the biologically relevant context of bacterial contamination in PCs. This approach was informed by our previous genomic analysis showing that PC-associated *S. aureus* strains exhibit altered virulence and secretion profiles, and by our earlier demonstration that SEG and SEH are secreted and detectable in PCs during storage [18,19]. Using the wildtype strain and SE-deficient mutants we assessed the net SE-dependent effects under conditions that mirror transfusion contamination. Data presented herein revealed a multifaceted effect of *S. aureus* contamination on platelet activation and function during PC storage, implicating both classical platelet activation pathways and staphylococcal toxin-dependent modulation. First, the progressive increase in CD62P (P-selectin) and Annexin V exposure over days 0 to 5 in all conditions underscores that platelets undergo baseline storage-induced stress and activation; however, PCs contaminated with wildtype *S. aureus* exhibited a significantly accelerated or amplified activation relative to non-spiked controls. The mitigated activation observed with SE-deficient mutants PC-Δ*seg*, PC-Δ*seh* and PC-ΔΔ*segh* show that SEs contribute quantitatively to platelet activation. Although the precise mechanisms by which SEs influence platelet activation remain to be fully defined, several biologically plausible pathways may underlie the responses observed in this study. Platelets express functional pattern-recognition receptors, including TLR2 and TLR4, which have been implicated in activation by bacterial components [29]. SE exposure could therefore engage TLR-dependent signaling and promote downstream inflammatory or activation cascades. Additionally, because SEs act as superantigens in immune cells and bind MHC-II on antigen-presenting cells, it is conceivable that SEs could initiate analogous MHC- or superantigen-like interactions on platelet surfaces, contributing to activation or granule release. Intracellular signaling pathways such as cAMP modulation, MAPK phosphorylation, and related kinase networks may also be involved, as these pathways are known regulators of platelet activation dynamics [30]. Furthermore, our observations are consistent with prior reports that *S. aureus* can directly activate platelets via bacterial adhesins and fibrinogen bridging (e.g., ClfA, FnBPA) or coagulation cascade engagement [9,10], and indirectly via superantigen toxins like TSST-1 and staphylococcal enterotoxin type B (SEB) [12,17]. Like the other classical SEs, SEG and SEH potentially cause indirect activation of platelet via the well-established superantigenic pathway. In particular, the ability of *S. aureus* to accelerate platelet reactivity has been observed in infective endocarditis patients (compared to coagulase-negative staphylococci), further supporting that bacterial presence augments platelet hyperreactivity in vivo [31]. The fact that SE-deficient mutants show reduced activation implies a previously underappreciated role for enterotoxins in modulating platelet membrane changes or intracellular signaling.

In addition to platelet activation, our cytokine profiling results indicate that wildtype *S. aureus* contamination during PC storage triggers release of both anti- and pro-inflammatory mediators, including CCL2, EGF, TGF-β1, IL-8, IFN-γ, TNF-α, and MIP-3α. Interestingly, mutation of individual SEs altered the cytokine milieu: for example, SEG deletion increased levels of MIP-d1, TGF-β1, IFN-γ, and TNF-α relative to the wildtype baseline, while SEH deletion impacted EGF and IGF-1 expression. These findings suggest that SEs differentially regulate platelet-cytokine crosstalk in *S. aureus*-contaminated PCs. Although platelets are not traditionally seen as classical cytokine-producers, accumulating evidence supports their capacity to store or synthesize immunomodulatory mediators PF4 and TGF-β in response to stress or infection [32,33,34]. Our results extend this concept into the context of bacterial contamination of PCs. The toxin-dependent modulation of cytokines may also reflect indirect effects.

Complementing our studies on platelet activation and cytokine profile, we presented miRNA sequencing and validation data providing a novel insight into how *S. aureus* and SEs reshape platelet regulatory networks under stress. We observed stable baseline expression of highly abundant miRNAs (let-7 family, miR-486-5p), serving as internal reference points. Previous studies report constitutive expression throughout PC storage for miRNAs belonging to the let-7 family [35,36]. Our study is the first to reveal constant baseline expression for circulating miR-486-5p in stored PCs. This miRNA has been implicated in several signaling pathways and is suggested to play a key role in non-malignant diseases [37]; therefore, miR-486-5p may serve as a potential marker for assessing the platelet viability in PCs. Additionally, the transient upregulation observed for miRNAs like miR-221-3p, miR-26a-5p, miR-98-5p, and miR-21-5p, when comparing PCs contaminated with *S. aureus* with PC control, is a hallmark of a temporally restricted adaptive or stress response [38]. The brief spike, peaking around day 3 and declining by day 5, probably allows cells to rapidly adjust their gene expression to cope with stressors from the PC storage environment. Each of these specific miRNAs targets different cellular pathways to facilitate survival, reduce damage, and promote recovery. For instance, research indicates that miR-98-5p has protective functions, particularly against oxidative stress and apoptosis [39], and miR-221-3p is associated with regulating inflammation and cell proliferation, which are crucial processes during a stress response [40]. Detecting high levels of these miRNAs at day 3 of storage could indicate bacterial contamination of PCs. Furthermore, differential expression of miRNAs including miR-98-5p, miR-146a-5p, miR-151a-5p, miR-221-3p, and miR-320a-3p between wildtype and SE-mutant inoculated PC samples further indicates that SEs modulate platelet miRNA responses. For instance, miR-320a-3p is known to be abundant in prolonged stored PCs [35], its down regulation in *S. aureus* wildtype PCs and markedly elevated expression (~3 to 6 folds) in SE-mutant PCs, together with altered expression of miR-221 and miR-146a, implicate SEs in suppression or fine-tuning of inflammatory miRNA networks in platelets. These miRNAs are known to regulate immune and inflammatory pathways and have been implicated in sepsis and bacterial infection settings (modulation of NF-κB, TLR signaling) [41,42,43]. Indeed, aberrant platelet miRNA expression has been correlated with sepsis severity (miR-26b) [44], further supporting the biological relevance of our findings.

Importantly, the functional virulence assay in silkworm larvae reinforces the biological significance of our in vitro observations showing that contaminated PCs harboring wildtype *S. aureus* exerted significantly higher lethality (lower LD_50_) compared to SE-deletion mutants. We have previously demonstrated that *S. aureus* CBS2016-05 increases mortality rate of silkworms [45] and this study revealed that the presence of SEs in PCs contributes to virulence in silkworm larvae, possibly by modulation of the host–pathogen interactions. The latter merits further investigation in future work. This connection between platelet-dependent modulation and in vivo virulence underscores the translational relevance of our in vitro observations.

**Figure 4:**
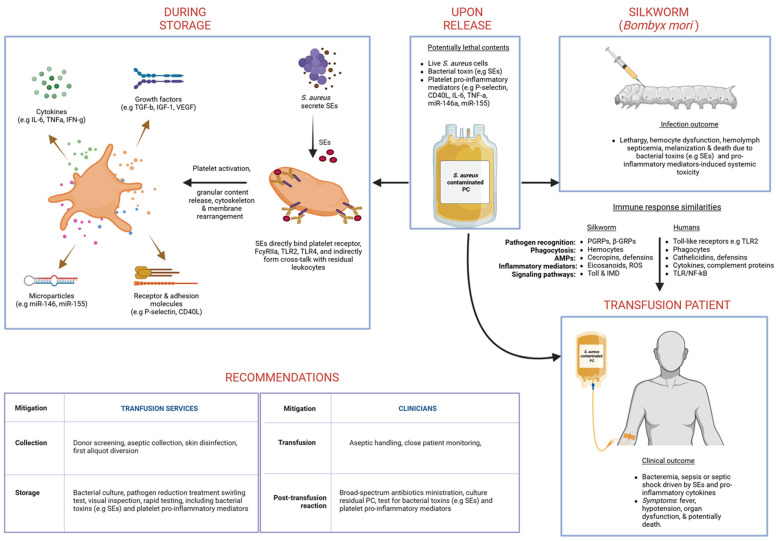
Summary of findings and significance in transfusion medicine. This figure was created using © 2025 BioRender (https://BioRender.com).

Altogether (Figure 4), our integrated dataset indicates that (i) *S. aureus* contamination accelerates platelet activation and procoagulant properties during PC storage; (ii) SEs modulate platelet-driven cytokine release and miRNA regulatory networks; and (iii) these molecular alterations can influence bacterial virulence outcomes. From a transfusion safety perspective, this work highlights that SEs may exacerbate platelet damage or prothrombotic potential and raise the possibility of miRNA or cytokine signatures as early biomarkers of contaminated PCs, which could be incorporated into screening protocols as early indicators of bacterial contamination or SE activity. Furthermore, inhibiting SEs either directly or as part of enhanced pathogen reduction strategies may mitigate platelet activation and inflammatory responses, thereby improving the efficacy and safety of PC transfusions.

Nonetheless, some limitations of our studies should be considered and merit-mention. First, while we show association between SE presence and miRNA/cytokine modulation, the mechanistic pathways remain to be delineated; for example, whether SEs act via receptor binding on platelets, indirect plasma mediators, or via platelet internalization. Second, our silkworm model, while convenient with rapid infection kinetics, ethical simplicity and suitability for high-throughput virulence screening, is an invertebrate proxy and does not fully recapitulate human pathophysiology. For example, silkworms rely solely on innate immunity and lack adaptive immune components such as T cells, B cells, and antibody-mediated responses [46]. Moreover, several mammalian-specific pathways involved in inflammation, cytokine signaling, and platelet–pathogen interactions are not fully represented in invertebrate hosts. As such, our findings from the silkworm killing assay only provide an indication of relative virulence rather than a direct surrogate for human pathophysiology. Future work should test mammalian models or human cell-based systems to dissect signaling pathways like TLRs, inflammasome, MAPK, and evaluate candidate miRNA or protein biomarkers for detection of bacterial contamination in PCs. Finally, this research was performed using wildtype and SE-deficient strains to assess the net SE-dependent effects under conditions that mirror transfusion contamination; therefore, we acknowledge that other bacterial components may contribute to the observed responses; however, this whole-bacterium model provides essential ecological validity. Future studies will complement these findings using purified SEG and SEH to define direct toxin-specific mechanisms and to delineate direct versus synergistic toxin effects.

## 4. Conclusions

In summary, contamination of PCs with *S. aureus* markedly intensifies platelet activation and procoagulant phenotypes during storage, and staphylococcal enterotoxins further modulate platelet-driven cytokine secretion and miRNA regulatory networks. These perturbations correlate with enhanced bacterial virulence in vivo, emphasizing the dual threat of bacterial contamination of PCs: direct pathogen burden and platelet-mediated exacerbation of pathogenicity. Our findings suggest that targeting enterotoxin-mediated signaling, or monitoring miRNA/cytokine signatures, may provide novel strategies for early detection or mitigation of contaminated PCs. Further mechanistic and translational validation is warranted to harness these insights toward safer transfusion practices.

## 5. Materials and Methods

### 5.1. Staphylococcus aureus Isolates and Inoculation of PCs

*S. aureus* strain CBS2016-05 [2] and derivation SE-mutants (CBS2016-05Δ*seg,* CBS2016-05Δ*seh* and CBS2016-05ΔΔ*segh*) used in this study were generated as previously described [14]. PC units were manufactured at the Canadian Blood Services NetCAD Facility (Vancouver, BC, Canada) in agreement with standard procedures. The PCs were shipped to the Canadian Blood Services Microbiology laboratory in Ottawa, Canada.

**Figure 5:**
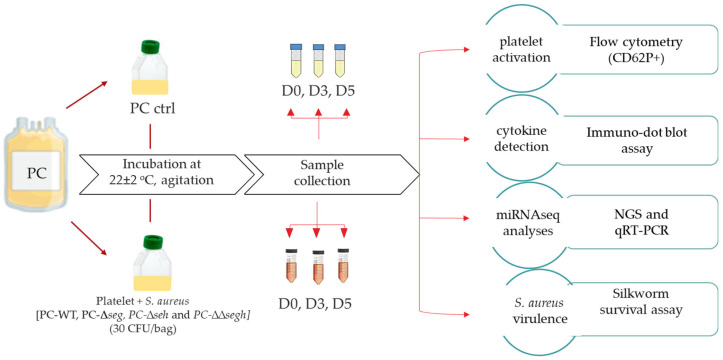
Experimental approach.

The experimental approach of the study is described in Figure 5. PCs were spiked with *S. aureus* CBS2016-05 wildtype (PC-WT), CBS2016-05Δ*seg* (PC-Δ*seg*), CBS2016-05Δ*seh* (PC-Δ*seh*), and CBS2016-05ΔΔ*segh* (PC-ΔΔ*segh*) at an initial inoculum of 30 CFU/PC unit. This study is premised on our previous findings on PCs contaminated with the same *S. aureus* wildtype strain used in this current study, which demonstrate that SEG and SEH are expressed at both RNA and protein levels in PCs during storage [18,19]. Non-spiked PC (PC-Ctrl) was used as control. Both PC-Ctrl and *S. aureus* inoculated PCs were incubated under PC storage conditions at 20 ± 2 °C with gentle agitation. Samples were collected at different timepoints (days 0, 2, 3 and 5) for downstream analyses. Day 0 refers to the timepoint when PCs were inoculated with *S. aureus* and is the baseline sampling time immediately after bacterial contamination. All subsequent sampling timepoints (e.g., day 1, day 2) represent timepoints relative to this baseline. These experiments were repeated for three biological replicates.

### 5.2. Flow Cytometric Analyses of P-Selectin and Annexin V Expression

Platelet activation was assessed using an Attune™ Flow Cytometer (Thermo Fisher Scientific, Mississauga, ON, Canada) following standard instrument setup and sample staining procedures. PC samples inoculated with wildtype *S*. *aureus*, (PC-WT), PC-Δ*seg*, PC-Δ*seh*, and PC-ΔΔ*segh*, as well as PC-Ctrl were analyzed at storage days 0, 3 and 5, for activation markers CD62P and phosphatidylserine residue (Annexin V binding), with CD41a serving as a platelet identifier. Platelet counts were obtained from Sysmex cell counter data provided by NetCAD, and working suspensions were prepared at a concentration of 2 × 10^6^ platelets per 100 μL. For the CD41a + CD62P panel, platelets were diluted in phosphate-buffered saline (PBS), while for the CD41a + Annexin V panel, platelets were suspended in 1× Annexin binding buffer prepared (Thermo Fisher Scientific, Mississauga, ON, Canada). Fluorescence-minus-one (FMO) and isotype controls (for CD41a + CD62P panel only) were included for gating optimization using non-spiked control samples. Staining with FITC-conjugated anti–P-selectin antibody (BD Biosciences, Mississauga, ON, Canada) and Annexin-V-APC (BD Pharmingen, San Jose, CA, USA) was performed in the dark at room temperature for 20 min, followed by the addition of 500 μL PBS to each sample prior to measurements. Forward scatter (FSC) and side scatter (SSC) parameters were used to gate platelet populations, and fluorescence signals were collected using appropriate channels for APC and Alexa Fluor 488. Data were analyzed to determine the proportion of CD41a-positive platelets positive for the expression of activation markers CD62P and Annexin V.

### 5.3. Platelet Cytokine Detection and Quantification

PCs were sampled for cytokine detection at day 0, 2 and 3 of storage, and centrifuged at 10,000 rpm for 10 min at 4 °C before freezing the supernatant at −80 °C. Frozen PC supernatants were thawed on ice, centrifuged at 6500 rpm for 5 min at 4 °C, and the clear supernatants were analyzed using a membrane-based cytokine antibody array kit and manual (RayBio^®^ C-Series Human Cytokine Antibody Array C5, R&D Systems, Norcross, GA, USA) as per the manufacturer’s manual. Briefly, the array membranes were first blocked with blocking buffer for 30 min at room temperature, followed by incubation with the PC supernatant samples diluted 1:2, for 5 h at room temperature or overnight at 4 °C under gentle rocking. Following incubation, membranes were washed three times and further incubated with biotinylated antibody cocktail for 2 h at room temperature or overnight at 4 °C, washed as above, and then incubated with HRP–streptavidin (1:1000 dilution) for 2 h at room temperature. After a final wash, chemiluminescent signals were developed by adding Detection Buffers and visualized using a chemiluminescence imaging system (ChemiDoc, Hercules, CA, USA). Upon completion of the protocol, the cytokine dot blot membranes were analyzed on UN-SCAN-IT Gel Analysis Software, v 7.1 (Silk Scientific, UT, USA, 2025, Provo, UT, USA) measuring the dot signal intensities in pixels. The mean pixel value for each expressed cytokine was considered after deducting the background signal and mean value of the negative control spots in each membrane analyzed. For relative cytokine expression quantification, samples collected at different storage timepoints of the same PC were normalized to day 0 or the fresh PC. Then, the same PC spiked individually with *S. aureus* wildtype and derivative SE mutants were compared relative to the non-spiked PC as background control.

### 5.4. MicroRNA Next Generation Sequencing and Differential Expression Analysis

#### 5.4.1. Platelet miRNA Extraction from PCs

miRNA was extracted from PC-WT and PC-Ctrl samples collected at days 0, 2, 3 and 5 using the miRNeasy Serum/Plasma Advanced Kit (Qiagen, Toronto, ON, Canada) according to the manufacturer’s protocol, with synthetic cel-miR-39 RNA spike-in (Qiagen, Toronto, ON, Canada) added as an exogenous control. Briefly, 400 μL of thawed PC was lysed with Buffer RPL, mixed with Buffer RPP, and centrifuged to remove precipitates. The clarified lysate was combined with isopropanol, and RNA was purified through successive washes with Buffers RWT, RPE, and 80% ethanol. The RNA pellet was air-dried and eluted in 20 μL nuclease-free water. RNA yield and quality were assessed using BioAnalyzer and Qubit fluorometric quantification.

#### 5.4.2. Small RNA Library Preparation and Sequencing

Small RNA libraries were prepared using the Lexogen Small RNA-Seq Library Prep Kit (Lexogen, Vienna, Austria) following the manufacturer’s instructions. Briefly, adapter ligation, reverse transcription, and PCR amplification were performed to generate indexed (SRi7001-SRi7024) libraries, which were purified using magnetic beads. Library quality was confirmed by fragment analysis, and sequencing was performed on an Illumina platform at StemCore Laboratories (Ottawa, ON, Canada).

#### 5.4.3. Bioinformatic Analysis

Sequencing data were retrieved from Illumina BaseSpace and processed using the nf-core/smrna pipeline v2.2.4 [47]. Reads were aligned to the human mature and hairpin miRNA reference sequences from miRBase [48] using Bowtie [49]. Count matrices were generated, normalized and differential expression analyzed with DESeq2 [50], applying fold change shrinkage with apeglm [51].

### 5.5. Quantitative Real-Time PCR of Validation of miRNA Data

MiRNAs extracted from PC samples spiked with wildtype *S. aureus*, (PC-WT), PC-Δ*seg*, PC-Δ*seh*, and PC-ΔΔ*segh*, as well as PC-Ctrl at days 0, 3 and 5, were analyzed using the miRCURY LNA Custom PCR Assay (Qiagen, Toronto, ON, Canada) following the manufacturer’s protocol. Briefly, reverse transcription was performed using the miRCURY LNA RT Kit to polyadenylate and convert miRNA into cDNA. The resulting cDNA was amplified using the miRCURY LNA SYBR Green PCR Master Mix and custom LNA primers specific to selected miRNAs (miR-146a, miR-98, miR-191, miR-221, miR-320a, miR-103a, miR-155a, and miR-151a) (Qiagen, Toronto, ON, Canada). Reactions were run in duplicate on a CFX96 Touch™ Real-Time PCR Detection System (Bio-Rad, Hercules, CA, USA). MiR-191 and miR-103a served as positive controls, and nuclease-free water as the negative control. Quantification cycle (Cq) values were used to calculate relative transcript copy numbers following melt curve analysis.

### 5.6. Silkworm Survival Assay

Silkworm survival assay was performed according to procedures reported in Kumaran and Ramirez-Arcos, 2025 [45]. Briefly, *Bombyx mori* eggs (obtained from Coastal Silk) were hatched at room temperature, and larvae were reared on artificial silkworm chow (Coastal Silk) supplemented with 300 mg/kg vancomycin. Fifth-instar silkworm larvae (day 1–2 post-molt) were transferred to vancomycin-free chow for at least 24 h before experimental use. For lethal dose 50 (LD_50_) bacterial load determination, *S. aureus* CBS2016-05 wildtype and derivative mutant strains PC-Δ*seg*, PC-Δ*seh,* PC-ΔΔ*segh* were grown to stationary phase in trypticase soy broth medium at 37 °C and resuspended in insect saline (0.6% NaCl). Ten-fold serial dilutions were injected (30 µL per larva) into the hemolymph of ten larvae per dilution. Mortality was recorded over 72 h at 37 °C, with death defined as lack of movement upon probing. Insect saline–injected larvae served as controls. Bacterial loads were confirmed by plating on trypicase soy agar, and LD_50_ values were calculated by probit regression using MedCalc v23.2.6 (MedCalc Software, Ostend, Belgium). Subsequently, the above wildtype and SE-mutant strains were spiked into PCs and incubated for 5 days at 20 ± 2 °C/agitation. 30 µL of PCs containing bacterial loads corresponding to the respective established LD_50_ values, were used to inject groups of ten larvae as reported above. Kaplan–Meier survival analysis was performed.

### 5.7. Statistical Analyses

All experiments were performed in triplicate unless otherwise stated. Data were analyzed using GraphPad Prism (version 9.0.0). Quantitative results are expressed as mean ± standard deviation (SD). One-way ANOVA followed by Tukey’s multiple comparison test was used to assess differences in CD62P and Annexin V expressions among groups (PC-Ctrl, PC-WT, PC-Δ*seg*, PC-Δ*seh*, and PC-ΔΔ*segh*) across storage days (0, 3, and 5). Cytokine array densitometry data were analyzed using two-way ANOVA to evaluate the effects of bacterial strain and storage time. DESeq2 was used for differential expression analysis of miRNA sequenced data. miRNAs with fold change ≥ 2 and adjusted *p* < 0.05 (FDR corrected) were considered significantly differentially expressed. The ^ΔΔ^Ct method and Student’s *t*-test were employed for RT-qPCR data. Silkworm virulence assays were assessed using Kaplan–Meier survival analysis. LD_50_ values were calculated using nonlinear regression. Differences were considered statistically significant at *p* < 0.05.

## Figures and Tables

**Table 1:** Cytokines released in *S. aureus* CBS2016-05 contaminated PCs (PC-SA) versus PC control (PC-Ctrl). N = 3.

Cytokine Mean (Dot Pixel Range)	Day 0		Day 2		Day 3	
	PC-Ctrl	PC-SA	PC-Ctrl	PC-SA	PC-Ctrl	PC-SA
IL-8	15.83–20.01	16.34–25.68	18.24–28.66	55.45–58.46	31.21–45.79	81.91–98.29
IL-3	2.19–3.09	1.75–2.09	3.82–4.09	7.19–11.4	4.86–6.11	8.28–12.41
CCL2	18.64–19.85	19.11–21.80	22.01–25.85	31.52–34.66	26.48–31.36	22.22–32.39
EGF	15.97–20.37	13.53–18.81	35.22–40.49	62.29–69.39	53.6–61.53	96.59–103.33
MIP-d1	31.73–35.11	31.38–34.58	32.41–38.15	58.17–62.53	43.96–44.97	78.17–84.63
TGF-β1	2.30–4.41	4.10–4.50	8.26–12.89	44.4–58.00	10.34–14.71	34.82–37.75
MIP-3α	1.97–2.70	2.95–3.64	4.92–5.87	14.52–16.65	6.96–11.72	20.95–27.41
IFN-γ	2.53–4.92	2.73–3.16	6.57–9.81	17.35–26.18	29.15–39.79	40.04–60.40
IGF-1	0.82–1.95	0.69–1.96	7.71–9.63	12.75–13.49	10.11– 13.71	30.7–31.08
TNF-α	1.91–3.48	0.45–1.25	1.25–3.96	49.78–62.12	27.01–35.34	62.88–76.85
IL-6	1.23–5.24	2.61–1.26	6.72–8.35	10.06–12.85	5.98–7.47	4.70–6.11
CXCL1	2.42–3.89	1.54–3.09	4.90–6.99	8.01–9.83	7.04–10.86	19.54–22.4
G-CSF	3.86–4.82	1.39–4.20	3.86–5.31	8.24–11.09	6.17–7.82	16.25–20.99
CXCL13	3.69–4.39	1.77–2.03	5.10–6.29	10.64–12.06	6.29–8.23	15.29–17.91
OPN	68.12–74.04	82.95–97.41	60.47–69.01	128.51–137.64	156.36–157.52	151.69–195.98

**Table 2:** Silkworm killing assay. Increase survival of silkworms injected with PCs inoculated with SE mutants. SEs heighten *S. aureus* virulence. * Significant difference between LD_50_ of WT and ∆∆*segh* (*p* < 0.05) (*n* = 3).

*S. aureus* Strain	PC-WT	PC-Δ*seg*	PC-Δ*seh*	PC-Δ*segh*
LD_50_ [CFU/larvae]	~3.31 × 10^6^	~2.31 × 10^7^	~3.51 × 10^7^	~8.9 × 10^7^ *
Silkworm survival from injection with *S. aureus*-spiked PCs (%)	27	37	30	40 *

## Data Availability

The original contributions presented in this study are included in the article and Supplementary Material. Further inquiries can be directed to the corresponding author.

## Supplementary Materials

The following supporting information can be downloaded at: https://www.mdpi.com/article/10.3390/toxins17120593/s1, Figure S1: 15 most highly expressed miRNA in non-spiked platelet concentrates (PC-Ctrl) during storage from days 0 to day 5 at 22 ± 2 °C under gentle agitation; Figure S2: 15 most highly expressed miRNA in PCs contaminated with *S. aureus* strain CBS2016-05 wildtype (PC-WT) during storage from days 0 to day 5 at 22 ± 2 °C under gentle agitation.

## Abbreviations

The following abbreviations are used in this manuscript:

ANOVA	Analysis of Variance
CCL2	C-C Motif Chemokine Ligand 2
CD62P	Cluster of Differentiation 62P (P-selectin)
CXCL9	C-X-C Motif Chemokine Ligand 9
ΔΔ*segh*	Double SEG/SEH-deficient mutant strain
Δ*seg*	SEG-deficient mutant strain
Δ*seh*	SEH-deficient mutant strain
EGF	Epidermal Growth Factor
FDR	False Discovery Rate
IFN-γ	Interferon gamma
IGF-1	Insulin-like Growth Factor 1
IL-8	Interleukin 8
LD_50_	Median Lethal Dose
MAPK	Mitogen-Activated Protein Kinase
miRNA	MicroRNA
MIP-3α	Macrophage Inflammatory Protein 3 alpha
NF-κB	Nuclear Factor Kappa-light-chain-enhancer of Activated B cells
OPN	Osteopontin
PBS	Phosphate-Buffered Saline
PC	Platelet Concentrate
RT-qPCR	Reverse Transcription Quantitative Polymerase Chain Reaction
*S. aureus*	*Staphylococcus aureus*
SD	Standard Deviation
SE	Staphylococcal Enterotoxin
SEG	Staphylococcal Enterotoxin G
SEH	Staphylococcal Enterotoxin H
TGF-β1	Transforming Growth Factor beta 1
TLR	Toll-like Receptor
TNF-α	Tumor Necrosis Factor alpha
