# Supplementary material for: Staphylococcal Enterotoxins Modulate Platelet Response During Storage of Platelet Concentrates and Impair Silkworm Survival
Source: Toxins (Basel). 2025 Dec 11;17(12):593. doi: 10.3390/toxins17120593 (PMC12737686; doi:10.3390/toxins17120593)
Supplement: Supplementary file 1 [file toxins-17-00593-s001.zip › toxins-3991242-supplementary.pdf]

Supplementary Materials: Staphylococcal Enterotoxins Modulate Platelet Response During Storage of Platelet Concentrates and Impair Silkworm Survival

Sylvia Ighem Chi, Chelsea McGregor, Nicolas Pineault and Sandra Ramirez-Arcos

Day 0	Day 2	Day 3	Day 5
hsa-let-7a-5p	hsa-let-7a-5p	hsa-miR-486-5p	hsa-miR-486-5p
hsa-let-7f-5p	hsa-let-7f-5p	hsa-let-7a-5p	hsa-let-7f-5p
hsa-miR-486-5p	hsa-miR-486-5p	hsa-let-7f-5p	hsa-let-7a-5p
hsa-miR-98-5p	hsa-miR-26a-5p	hsa-let-7i-5p	hsa-miR-26a-5p
hsa-miR-10a-5p	hsa-let-7b-5p	hsa-let-7b-5p	hsa-let-7i-5p
hsa-let-7i-5p	hsa-miR-21-5p	hsa-miR-26a-5p	hsa-let-7g-5p
hsa-let-7b-5p	hsa-let-7i-5p	hsa-miR-21-5p	hsa-let-7b-5p
hsa-miR-26a-5p	hsa-miR-103a-3p	hsa-miR-98-5p	hsa-miR-221-3p
hsa-let-7g-5p	hsa-miR-103b	hsa-let-7g-5p	hsa-miR-103b
hsa-miR-191-5p	hsa-let-7g-5p	hsa-miR-103a-3p	hsa-miR-103a-3p
hsa-let-7c-5p	hsa-miR-221-3p	hsa-miR-103b	hsa-miR-98-5p
hsa-let-7d-5p	hsa-miR-10a-5p	hsa-miR-221-3p	hsa-miR-146a-5p
hsa-miR-21-5p	hsa-miR-98-5p	hsa-miR-25-3p	hsa-miR-21-5p
hsa-let-7e-5p	hsa-miR-146a-5p	hsa-miR-10a-5p	hsa-miR-10a-5p
hsa-miR-103a-3p	hsa-miR-25-3p	hsa-let-7e-5p	hsa-miR-25-3p

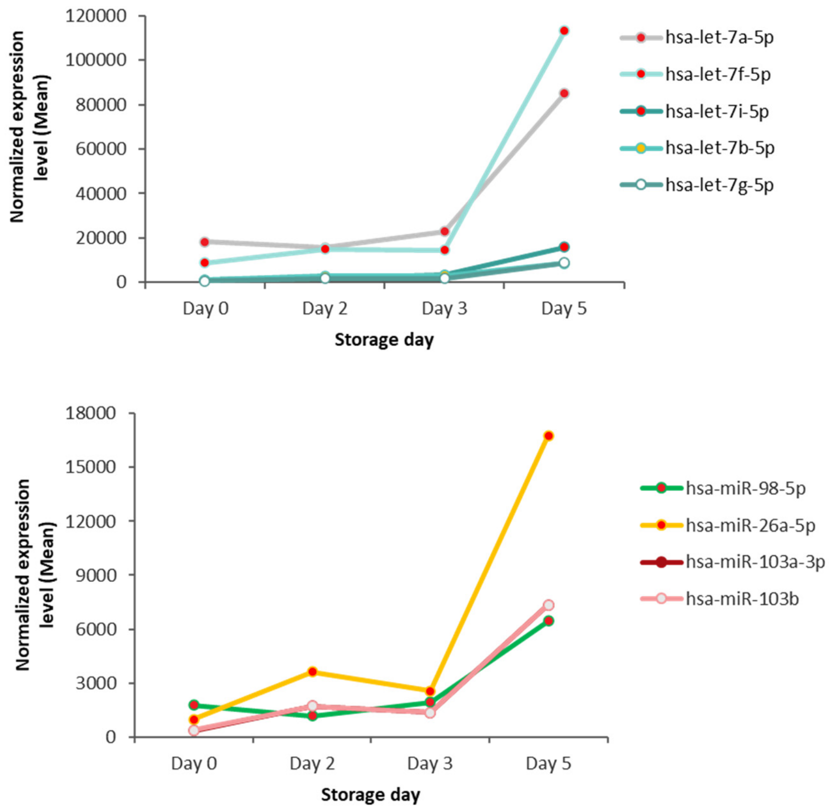

<u>Figure S1</u>: 15 most highly expressed miRNA in non-spiked platelet concentrates (PC-Ctrl) during storage from days 0 to day 5 at 22±2 °C under gentle agitation

 https://doi.org/10.3390/toxins17120593

Day 0	Day 2	Day 3	Day 5
hsa-miR-486-5p	hsa-miR-486-5p	hsa-let-7a-5p	hsa-let-7a-5p
hsa-let-7a-5p	hsa-let-7f-5p	hsa-let-7f-5p	hsa-let-7f-5p
hsa-let-7f-5p	hsa-let-7a-5p	hsa-let-7i-5p	hsa-miR-486-5p
hsa-let-7i-5p	hsa-miR-26a-5p	hsa-let-7b-5p	hsa-let-7b-5p
hsa-let-7b-5p	hsa-let-7i-5p	hsa-let-7g-5p	hsa-let-7i-5p
hsa-miR-98-5p	hsa-let-7b-5p	hsa-miR-98-5p	hsa-miR-98-5p
hsa-miR-21-5p	hsa-miR-21-5p	hsa-miR-486-5p	hsa-let-7g-5p
hsa-let-7g-5p	hsa-miR-103a-3p	hsa-miR-221-3p	hsa-miR-221-3p
hsa-miR-26a-5p	hsa-let-7g-5p	hsa-miR-26a-5p	hsa-miR-26a-5p
hsa-miR-221-3p	hsa-miR-98-5p	hsa-miR-103a-3p	hsa-miR-103a-3p
hsa-miR-103a-3p	hsa-miR-143-3p	hsa-miR-103b	hsa-miR-103b
hsa-miR-103b	hsa-miR-92a-3p	hsa-let-7d-5p	hsa-let-7c-5p
hsa-let-7c-5p	hsa-miR-25-3p	hsa-let-7c-5p	hsa-miR-409-3p
hsa-miR-10a-5p	hsa-miR-103b	hsa-miR-92a-3p	hsa-miR-146a-5p
hsa-miR-92a-3p	hsa-miR-221-3p	hsa-miR-25-3p	hsa-let-7d-5p

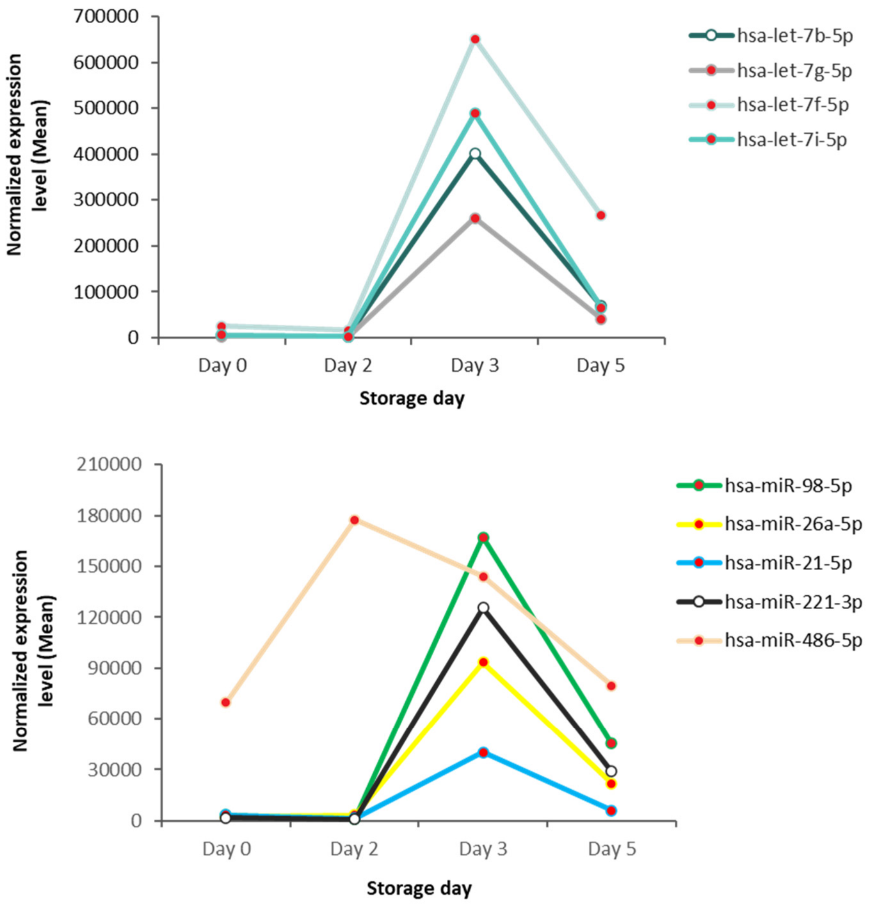

Figure S2: 15 most highly expressed miRNA in PCs contaminated with wildtype S. aureus CBS2016-05 (PC-WT) during storage from days 0 to day 5 at 22±2 oC under gentle agitation

Table S1: Ten differentially expressed microRNAs in platelet concentrate (PC), according to days of storage.

MiRNA sequencing report										
miRBase Database			Deep sequencing (Raw data)							
Platelet miRNA	Mature sequence	nt	Day 0		Day 2		Day 3		Day 5	
			PC Ctrl	PC-WT	PC Ctrl	PC-WT	PC Ctrl	PC-WT	PC Ctrl	PC-WT
hsa-let-7a-5p	UGAGGUAGUAGGUUGUAUAGUU	22	54557	97068	46579	43661	78552	2967813	254793	467663
hsa-let-7b-5p	UGAGGUAGUAGGUUGUGUGGUU	22	3489	10365	8652	11072	8904	401634	25482	69362
hsa-let-7f-5p	UGAGGUAGUAGAUUGUAUAGUU	22	26438	75581	44590	69661	53547	651306	113161	266854
hsa-miR-98-5p	UGAGGUAGUAAGUUGUAUUGUU	22	5276	10231	3499	4951	7758	167233	19352	45337
hsa-miR-146a-5p	UGAGAACUGAAUUCCAUGGGUU	22	1352	4330	4084	4118	7720	21618	15853	12445
hsa-miR-221-3p	AGCUACAUUGUCUGCUGGGUUUC	22	1389	8326	4666	4917	5871	125535	43992	28927
hsa-miR-320a-3p	AAAAGCUGGGUUGAGAGGGCGA	22	1291	4190	3871	3522	4058	34126	6171	10728
hsa-miR-151a-5p	UCGAGGAGCUCACAGUCUAGU	22	4033	4518	3442	3664	5445	20488	7700	8788
hsa-miR-26a-5p	UUCAAGUAAUCCAGGAUAGGCU	22	4124	7475	10858	9960	7656	93320	16773	21888

hsa-miR-21-5p	UAGCUUAUCAGACUGAUGUUGA	22	3344	9869	7422	5464	5919	40192	21530	18435
